# Infectious vaginitis among women seeking reproductive health services at a sexual and reproductive health facility in Kampala, Uganda

**DOI:** 10.1186/s12905-023-02835-w

**Published:** 2023-12-19

**Authors:** Huzaima Mujuzi, Aggrey Siya, Rogers Wambi

**Affiliations:** 1 Department of Microbiology, UMC Victoria Hospital, Kampala, Uganda; 2 Department of Biosecurity, Ecosystems and Veterinary Public Health, Makerere University, P.O.Box 7060, Kampala, Uganda; 3 Department of Zoology, Entomology and Fisheries Sciences, Makerere University, P.O. Box 7060, Kampala, Uganda; 4 Department of Clinical Laboratories, Mulago National Referral Hospital, Kampala, Uganda

**Keywords:** Vulvo-vaginal candidiasis, Bacterial vaginosis, Trichomoniasis, Pruritus, Kampala, Uganda

## Abstract

**Background:**

Infectious vaginitis is one of the most prevalent conditions affecting women of reproductive age with significant clinical consequences. Bacterial vaginosis (BV), vulvo-vaginal candidiasis (VVC), and trichomoniasis (TV) are the main etiologies. Unfortunately, there is limited data on the prevalence and associated risk factors, especially in sub-saharan Africa. This study, thus, determined the prevalence and risk factors of infectious vaginitis among women seeking reproductive health services at a Marie-stopes health facility in urban areas of Kampala, Uganda.

**Methods:**

A cross-sectional study with 361 participants was conducted from July to October 2021. Data on risk factors and infection were collected via a structured questionnaire and laboratory analysis of vaginal swabs, respectively, with data analysis performed using Stata version 14.0 college station, Texas 77,845 US.

**Results:**

The ages of participants ranged from 18 to 49 years, with a mean age of 29.53 years. Overall, 58.45% were infected, of whom 33.24% had VVC, 24.93% had BV, and 0.28% had TV. Bivariate analysis revealed that women with pruritus (COR: 3.057, 95% CI: 1.940–4.819), pregnancy (COR: 4.914, 95% CI: 1.248–19.36), antibiotic use (COR: 1.592, 95% CI: 1.016–2.494), douching (COR: 1.719, 95% CI: 1.079–2.740), and multiple partners (COR: 1.844, 95% CI: 1.079–2.904) were more likely to have VVC, whereas having higher education status (University; Vocational) (COR: 0.325, 95% CI: 0.134–0.890; COR: 0.345, 95% CI: 0.116–0.905) reduced the risk. On the other hand, women with a smelly discharge (COR: 1.796, 95% CI: 1.036–3.110), IUD use (COR: 1.868, 95% CI: 1.039–3.358), and antibiotic use (COR: 1.731, 95% CI: 1.066–2.811) were more likely to have BV. Multivariable analysis identified pruritus (AOR: 2.861, 95% CI: 1.684–4.863) as the only independent predictor for VVC.

**Conclusion:**

Results indicate a high prevalence of infection among these women; therefore regular screening and treatment is recommended to curb the high rate of infection. More studies on risk factors of infection are recommended.

## Introduction

Infectious vaginitis refers to the inflammation and infection of the vagina [1]. It is mainly caused by bacterial vaginosis (BV), trichomoniasis (TV), and vulvo-vaginal candidiasis (VVC) which account for 90% of all etiologies among women of reproductive age [2]. There is public health concern about the serious sequelae of these infections, which include several gynecological and obstetrical disorders as well as amplification of sexually transmitted disease (STD) transmissions, particularly human immunodeficiency virus (HIV) [2–4].

Normally, the healthy vaginal tract of reproductive-aged women is colonized by commensal microflora, especially Lactobacillus, which maintains a balanced microbial ecosystem [5]. However, a shift in the lactobacilli population structure, either through the introduction of an organism or a disturbance, alters the homeostasis, resulting in the proliferation of pathogenic species and, subsequently, infectious vaginitis ensues [6].

Bacterial vaginosis, the most common cause of infectious vaginitis, is caused by a change in the complex balance of microflora in the vaginal tract in favor of anaerobic bacteria such as *Gardnerella vaginalis, Mobiluncus sp, and Mycoplasma sp* [7]. Vulva-vaginal candidiasis, the second common cause of infectious vaginitis, is caused by overgrowth of yeasts, especially *Candida albicans* [8], whereas trichomoniasis, the third common cause of infectious vaginitis, is a sexually transmitted disease caused by a flagellated parasite *Trichomonas vaginalis* [5].

In symptomatic women, these conditions are clinically characterized by malodor, irritation, pain, itching, and changes in color, consistency, and amount of vaginal discharge [2, 9, 10]. Several studies have identified a variety of risk factors for infectious vaginitis, including having multiple sexual partners, using hygiene products, smoking, pregnancy, antibiotic use, contraceptive methods, immunodeficiency, and diabetes, all of which can alter the vaginal ecosystem [5, 9–11]. Genetic predisposition, low socioeconomic status, and poverty are also linked with infection [5, 9–11].

Every year, 5–10 million women worldwide seek gynecological care for infectious vaginitis [12]. The global prevalence of BV ranges from 8 to 51%, depending on the population studied [5]. On the other hand, it’s estimated that about 75% of adult women will have at least one episode of VVC during their lifetime [3], whereas TV is estimated to affect between 57 and 180 million people worldwide every year [1]. In Africa, varying frequencies of these infections have been reported. In studies done in Cameroon, Ghana, Nigeria, and Ethiopia, overall prevalences of 52.45, 56.4%, 76%, and 15.4%, respectively, were reported for the three infections [1, 13–15].

In Uganda, there is insufficient data on the prevalence of these infections, particularly among women seeking reproductive health services in urban areas. Available data on the prevalence of infectious vaginitis has come from studies of women with other comorbidities in either a rural area [16] or among pregnant women attending antenatal clinics [17]. On the other hand, data on the risk factors for infectious vaginitis is still lacking in the country. Hence, there is a need to determine the prevalence and risk factors of infectious vaginitis among women seeking reproductive health services in urban areas of Kampala, Uganda. Understanding the prevalence and risk factors for infectious vaginitis is critical in designing effective interventions for women’s reproductive health.

## Materials and methods

### Study site, design

This was a cross-sectional study conducted among women seeking reproductive health services at a Marie-stopes health facility, at kavule in Kampala, Uganda, from July 12th to October 02nd, 2021. Marie-stopes is one of the largest sexual and reproductive health specialized service providers in the country, offering family planning, post-abortion care, maternity, STI management, laboratory services, and general consultation, among other services. According to the Ministry of Health grading, the health facility is an outpatient clinic with a functional laboratory and is comparable to a health center III. The facility employs 11 permanent staff members, including clinicians, midwives, laboratory technologists, sonographers, and other auxiliary workers. Every month, the health facility attends to an average of 800 women from Kampala city and the surrounding areas.

### Participant selection and sample size determination

Women for enrollment were chosen using a systemic sampling method in which the first woman was chosen at random and every sixth woman was chosen on consecutive days, Monday through Sunday. The sampling continued until the required sample size was realized. At 95% confidence, an assumed prevalence rate of 52.44% obtained from the study by Payne et al. [13], and 5% absolute precision, a sample size of 383 women was obtained for this study using the Kish Leslie’s formula (1965) [18]. All visiting females at this health facility who met the sampling criteria and gave their written informed consent to participate were enrolled in this study. Women with menstrual bleeding, those who declined to consent, or those who opted not to provide samples even after initially providing informed written consent, were excluded from this study. Out of 383 women approached for participation in this study, 370 gave consent and answered the questionnaire, however, only 361 of these provided samples. This study, therefore, considered a total of 361 women who provided samples and consent for the study.

### Data collection on socio-demographic, clinical manifestation, sexual behaviors, and practices of participants

Prior to administering the questionnaire, the research team shared the objectives of the study and clarified any issues raised by the participants. A structured questionnaire was administered to participants of this study to collect data on socio-demographic characteristics, lifestyle-related behaviors, routine hygienic practices, history of contraceptive intake, and clinical status of participants. Participants were assigned study identification numbers (IDs), which were used throughout the study. Communication was done in English and Luganda, the commonly used local language in Kampala.

### Sample collection, transportation, and laboratory investigations

Following a thorough explanation of the sampling procedure to the participants, a trained midwife obtained two sterile vaginal swabs from each participant. Each participant was placed in a lithotomic position, and the labia of the vagina was opened with a speculum, a sterile swab was gently inserted, and it was rotated around the lateral and posterior fornices of the vagina. The procedure was repeated to obtain two samples, which were labeled with study participants’ unique identifiers and placed in Amies transport medium from Delta lab, 300,287 amies viscose lot number 32,002,475. The samples were then immediately transported to the Microbiology department of UMC Victoria Hospital laboratory for analysis according to standard operating procedures. A wet preparation was prepared using one swab, and a gram smear was made using the other swab after it had been inoculated on a freshly prepared media plate.

For BV diagnosis, a Gram stain smear was prepared from one swab and examined microscopically, and the result was scored using Nugent’s scoring system (Nugent’s criteria) [19]. A participant was declared positive for BV if the Nugent score was between 7 and 10, but negative if it was less than 7. 10% of the smears were re-read by another reader as quality control, which was in agreement with the first reader.

TV was diagnosed with saline wet mounts on a glass slide with a coverslip, which was examined with X-100 and X-400 magnification. *T. vaginalis* motile trophozoites of round or oval shape were discovered. Their morphological identification on Giemsa-stained smears confirmed the diagnosis [20].

For the diagnosis of VVC, vaginal swab samples were inoculated on freshly prepared Sabouraud dextrose agar (SDA) plates infused with chloramphenicol and incubated at 37^0^ C for 48 to 72 h. This was followed by colony identification of creamy to white colonies and the appearance of yeasts on a Gram stain [11].

### Data management and analysis

The data was captured using a mobile electronic tool (ODK) and downloaded into an Excel worksheet (Microsoft® Excel for Windows, 2017). It was then edited and exported to Stata statistical package version 14.0 (College station, Texas 77,845 US) for analysis. Descriptive statistics were used to summarize the characteristics of the study participants. To summarize and present the data, frequencies, and proportions were used, while the prevalence of infection was calculated by dividing the number of women infected by the total number of participants. A bivariate analysis to determine the association between each of the three main causes of infectious vaginitis (BV, VVC, and TV) and the various independent variables was carried out using a simple logistic regression model through crude odds ratios (COR), *P*-values, and a 95% confidence interval (CI). A multivariable logistic regression model was developed to determine independent predictors of infection for all the independent variables included in the bivariate analysis with a *P*-value of less than 0.2 for each of the three main causes of infectious vaginitis through the adjusted odds ratio (AOR) and the corresponding 95% CI *P*-values of less than 0.05 were considered statistically significant.

### Ethical considerations

This study obtained ethical approval from the research and ethics committee of Mulago National Referral Hospital Research and Ethics Committee, number MHREC 2256. All methods were performed according to the relevant guidelines and regulations. The researcher asked for written informed consent from the potential participants. No participant was enrolled without their written informed consent. The names, identities, and information collected from the participants were treated with maximum confidentiality. A code was assigned to data that only the investigator can identify. The researcher behaved honestly, fairly, and respectfully to all participants who were involved in this study.

## Results

### Socio-demographic characteristics

The participants’ ages ranged from 18 years to 49 years, with a mean age of 29.53 years. More than two-thirds (72%) had attained a university level of education, more than a vocational level (22%), or a secondary level (5%). Among the seven occupations studied, sales and services (47%) were the most common, and the least common was unskilled manual labor (0.01%). More than half of the participants were married (60%) (Table 1).

**Table 1:** Showing Socio demographic characteristics of the participants

Variable	Category	N = 361	Percentage
**Age (Years)**	18–27	142	39.34%
	28–37	179	49.58%
	38–49	40	11.08%
**Education Level**	Secondary	19	5.26%
	University	261	72.30%
	Vocational	81	22.44%
**Occupation**	Agriculture	06	1.66%
	Housewife	17	4.71%
	Professional	57	15.79%
	Sales & services	170	47.09%
	Skilled manual	54	14.96%
	Student	54	14.96%
	Unskilled manual	03	0.83%
**Marital Status**	Yes	218	60.39%
	No	142	39.34%
	Divorced/ Widowed	01	0.28%
**Marriage duration**	0.1–10	178	49.31%
	11–20	40	11.08%
	Unmarried/Divorced	143	39.61

### Clinical characteristics

With regard to symptoms, the majority of participants (85.32%) reported at least one symptom. Among the symptoms, the majority of participants reported having pruritus (45.15%), followed by abnormal discharge (41.55%) and smelly discharge (21.05%). More than two-thirds of participants (80.61%) used one of the contraceptive methods, which included contraceptive pills (33.80%), IUDs (17.17%), and Depo Provera injections (7.76%). Only 2.77% of all participants were pregnant, and 11.63% had recently had an abortion or miscarriage. A third (36.84%) of the participants had recently used antibiotics, and 7% had a chronic illness, with diabetes (6.09%) being the most common (Table 2).

**Table 2:** Showing clinical characteristics of the participants

Variable	Category		N = 361	Percentage
**Symptoms**	Smelly vaginal discharge	Yes	76	21.05%
		No	285	78.95%
	Pruritus	Yes	163	45.15%
		No	198	54.85%
	Dyspareunia	Yes	17	4.71%
		No	344	95.29%
	Pain	Yes	39	89.20%
		No	322	10.80%
	Abnormal discharge	Yes	150	41.55%
		No	211	58.45
	None	Yes	53	14.68%
		No	308	85.32%
**Contraceptive method**	IUD	Yes	62	17.17%
		No	299	82.83%
	Implant	Yes	0	0.00%
		No	361	100.00%
	Condoms	Yes	27	7.48%
		No	334	92.52%
	Depo injection	Yes	28	07.76%%
		No	333	92.24%
	Emergency pills	Yes	34	9.42%
		No	327	90.58%
	Contraceptive pills	Yes	122	33.80%
		No	239	66.20%
	None	Yes	70	19.39%
		No	291	80.61%
**Abortion/miscarriage**	No		319	88.37%
	Yes		42	11.63%
**Currently Pregnant**	No		351	97.23%
	Yes		10	2.77%
**Chronic conditions**	Diabetes		22	6.09%
	HIV		05	1.39%
	None		334	92.52%
**Recent antibiotic use**	No		228	63.16%
	Yes		133	36.84%

### Behavioral characteristics

Among the behavioral characteristics, a third of the respondents (30.47%) douched regularly, using mostly soap (21.88%), more than three quarters (75.62%) had a single sexual partner while two-thirds (62.05%) reported 0–2 sexual encounters per week. Active smokers were only 5.26% in this study (Table 3).

**Table 3:** Showing Behavioral characteristics of the participants

Variable	Category	N = 361	Percentage
**Douching**	No	251	69.53%
	Yes	110	30.47%
	**Douching products**		
	Soap	79	21.88%
	Water	21	5.82%
	Vwash	07	1.94%
	Lawash	04	1.11%
	Garlic	04	1.11%
	Baking powder	03	0.83%
	Shama genital wash	02	0.55%
	Sodium bicarbonate	01	0.28%
	Feminine wash	01	0.28%
	None	239	66.20
**Sexual partners**	One	273	75.62%
	Multiple (≥ 2)	88	24.38%
**Sexual frequency**	0–2 times weekly	224	62.05%
	3–5 times weekly	137	37.95%
**Active smoker**	No	342	94.74%
	Yes	19	5.26%

### Prevalence of infectious vaginitis

Out of 361 women, 211 (58.45%) had infectious vaginitis, with 120 (33.24%) having VVC, 90 (24.93%) having BV, 1 (0.28%) having TV, and 21 (17.5%) having a mixed infection of BV and VVC (Table 4).

**Table 4:** Prevalence of Infectious vaginitis

Infection	N = 361	Positive samples	Prevalence (%)	95% CI
VVC	361	120	33.24	0.286–0.383
BV	361	90	24.93	0.207–0.297
TV	361	01	0.28	0.0004–0.0196
BV + VVC	361	21	17.5	0.156–0.334
VVC + BV + TV	361	211	58.45	0.172–0.220

Abbreviation: VVC-Vulvo-vaginal candidiasis, BV-bacterial vaginosis, TV-Trichomoniasis, 95% CI- 95% Confidence interval

**The association of socio-demographic factors, women’s practices, sexual behaviors, and clinical manifestations with the three main causes of infectious vaginitis (BV, VVC, and TV) among women seeking reproductive health services at a Marie-stopes health facility.**

In the bivariate analysis, higher education status (university degree and vocational level) was significantly associated with VVC (COR: 0.325, 95% CI: 0.134–0.890) (COR: 0.345, 95% CI: 0.116–0.905) and it was the only socio-demographic factor that was significantly associated with infection. Multivariable analysis, however, showed no significant association of demographic factors with any of the three main causes of infectious vaginitis. Associations of socio-demographic factors, women’s practices, sexual behaviors, and clinical manifestations with trichomoniasis could not be predicted in the modal due to its low prevalence (0.28%) in this study (Table 5).

**Table 5:** Association of socio-demographic characteristics with the three main causes of infectious vaginitis among women seeking reproductive health services at a Marie-stopes health facility

Variable	Bivariate analysis; VVC		Multivariable analysis; VVC		Bivariate analysis; BV		Multivariable analysis; BV	
Age	COR:95% CI	*P*-value	AOR: 95% CI	*P*-value	COR: 95% CI	*P*-value	AOR: 95% CI	*P*-value
18–27 Years	reference				reference			
28–37 Years	1.045, 0.655–1.666	0.854	-	-	1.044, 0.623–1.755	0.870	-	-
38–49 Years	0.404, 0.404–1.854	0.712	-	-	1.779, 0.834–3.793	0.136	-	-
**Education level**								
Secondary	reference				reference			
University	0.345, 0.134–0.890	0.028 *	0.358, 0.121–1.058	0.063	1.944, 0.550–6.867	0.302	-	-
Vocational	0.325, 0.116–0.905	0.032 *	0.340, 0.108–1.067	0.064	1.439, 0.375–5.521	0.596	-	-
**Occupation**								
Agriculture	reference				reference			
Housewife	0.833, 0113-6.111	0.858	-	-	0.667, 0.049–9.022	0.760	-	-
Professional	0.714, 0.118–4.308	0.714	-	-	2.703, 0.295–24.76	0.379	-	-
Skilled manual	0.919, 0.153–5.514	0.926	-	-	1.429, 0.152–13.43	0.755	-	-
Student	1.600, 0.270–9.490	0.605	-	-	1.279, 0.135–12.10	0.830	-	-
Sales & services	1.009, 0.180–5.673	0.992	-	-	1.746, 0.199–15.36	0.615	-	-
**Marital status**								
No	reference		reference		reference			
Yes	0.708, 0.453–1.105	0.128	0.864, 0.473–1.577	0.634	1.329, 0.808–2.186	0.263		-
**Duration**								
0.1–10	reference				reference		reference	
11–20	1.136, 0.544–2.369	0.521	-	-	1.827, 0.885–3.773	0.103	1.766, 0.842–3.704	0.132

Abbreviations: AOR: Adjusted odds ratio, COR: Crude odds ratio, 95% CI: 95% Confidence interval, (variables with a *P* value of less than 0.2 at bivariate analysis were entered into the multivariable analysis model).* Indicates significant association (*P* < 0.05)

**The association of reproductive history and clinical factors with the three main causes of infectious vaginitis among women seeking reproductive health services at Marie-stopes facility.**

In the bivariate analysis, having a smelly discharge was significantly associated with BV (COR: 1.796; 95% CI: 1.036–3.110) Similarly, IUD use (COR: 1.868, 95% CI: 1.039–3.358) was significantly associated with BV while pruritus (COR: 3.057; 95% CI: 1.940–4.819) and pregnancy (COR: 4.914; 95% CI: 1.248–19.36) were significantly associated with VVC. Multivariable analysis identified pruritus as the only independent predictor of VVC (AOR: 2.861, 95% CI: 1.684–4.863). The odds of women who presented with pruritus were 2.861 times more likely to have VVC than their counterparts. No independent predictor was identified for bacterial vaginosis. Associations of reproductive history and clinical factors with trichomoniasis could not be predicted in this modal due to its low prevalence (0.28%) in this study (Table 6).

**Table 6:** Association of reproductive history and clinical factors with the three main causes of infectious vaginitis among women seeking reproductive health services at a Marie-stopes health facility

Variable	Bivariate analysis; VVC		Multivariable analysis; VVC		Bivariate analysis; BV		Multivariable analysis; BV	
Smelly discharge	COR:95% CI	*P*-value	AOR: 95% CI	*P*-value	COR: 95% CI	*P*-value	AOR: 95% CI	*P*-value
No	reference		reference		reference		reference	
Yes	1.628, 0.968–2.739	0.066	1.385, 0.746–2.571	0.302	1.796, 1.036–3.110	0.037 *	1.520, 0.681–3.392	0.307
**Pruritus**								
No	reference		reference		reference		-	-
Yes	3.057, 1.940–4.819	< 0.001*	2.861, 1.684–4.863	< 0.001*	0.907, 0.561–1.466	0.689	-	-
**Dyspareunia**								
No	reference		reference		reference		-	-
**Yes**	2.361, 0.887–6.285	0.085	2.727, 0.913–8.148	0.072	1.688, 0.606–4.704	0.316	-	-
**Pain**								
No	reference		-	-	reference		-	-
Yes	0.881, 0.429–1.806	0.729	-	-	1.208, 0.575–2.538	0.617	-	-
**Abnormal discharge**								
No	reference		-	-	reference		-	-
Yes	1.300, 0.836–2.024	0.245	-	-	0.863, 0.530–1.406	0.554	-	-
**Asymptomatic**								
No	reference		reference		reference		-	-
Yes	0.311, 0.142–0.683	0.004 *	0.746, 0.300-1.653	0.528	0.759, 0.373–1.546	0.448	-	-
**Abortion/miscarriage**								
No	reference		-	-	reference		-	-
Yes	1.272, 0.654–2.475	0.478	-	-	1.078, 0.518–2.244	0.841	-	-
**Pregnant**								
No	reference		reference		reference		-	-
Yes	4.914, 1.248–19.36	0.023 *	3.625, 0.794–16.55	0.097	0.747, 0.156–3.585	0.716	-	-
**Diabetes**								
No	reference		-	-	reference		-	-
Yes	0.933, 0.370–2.354	0.884	-	-	0.879, 0.315–2.454	0.805	-	-
**Hiv**								
No	reference		-	-	reference		reference	
Yes	1.345, 0.222–8.157		-	-	4.638, 0.762–8.211		3.732, 0.582–23.93	0.165

Abbreviations: AOR: Adjusted odds ratio, COR: Crude odds ratio, 95% CI: 95% Confidence interval, (variables with a *P* value of less than 0.2 at bivariate analysis were entered into the multivariable analysis model).* Indicates significant association (*P* < 0.05)

**Association of practices and sexual behavior with the three main causes of infectious vaginitis (BV, VVC, and TV) among women seeking reproductive health services at a Marie-stopes health facility.**

In the bivariate analysis, antibiotics use was significantly associated with both BV and VVC, respectively (COR: 1.731, 95% CI: 1.066–2.811), (COR: 1.592; 95% CI: 1.016–2.494). Also having multiple (≥ 2) sexual partners (COR 1.844; 95% CI: 1.079–2.904), and regular douching (COR 1.719; 95% CI: 1.079–2.740) were significantly associated with VVC. However, in the multivariable analysis, no independent predictor was identified for any of the conditions. Associations of practices and sexual behavior with trichomoniasis could not be predicted in this modal due to its low prevalence (0.28) in this study (Table 7).

**Table 7:** Association of practices and sexual behavior with the three main causes of infectious vaginitis (BV, VVC and TV) among women seeking reproductive health services at a Marie-stopes health facility

Variable	Bivariate analysis; VVC		Multivariable analysis; VVC		Bivariate analysis; BV		Multivariable analysis; BV	
IUD use	COR:95% CI	*P*-value	AOR: 95% CI	*P*-value	COR: 95% CI	*P*-value	AOR: 95% CI	*P*-value
No	reference		reference		reference		reference	
Yes	1.451, 0.826–2.547	0.195	1.095, 0.574–2.088	0.782	1.868, 1.039–3.358	0.037 *	1.27, 0.559–2.889	0.568
**Condom use**								
No	reference		-	-	reference		-	-
Yes	1.005, 0.437–2.308	0.992	-	-	0.666, 0.244–1.813	0.426	-	-
**Emergency pills**								
No	reference		reference		reference		-	-
Yes	0.491, 0.207–1.163	0.106	0.456, 0.179–1.159	0.099	1.093, 0.490–2.439	0.827	-	-
**Contraceptive Pills**								
No	reference		-	-	reference		reference	
Yes	1.145, 0.723–1.814	0.564	-	-	0.596, 0.350–1.018	0.058	0.935, 0.457–1.911	0.853
**Antibiotic use**								
No	reference		reference		reference		reference	
Yes	1.592, 1.016–2.494	0.042 *	1.292, 0.774–2.155	0.326	1.731, 1.066–2.811	0.027 *	1.475, 0.781–2.789	0.231
**Sexual partners**								
0ne	reference		reference		reference		-	-
Multiple	1.770, 1.079–2.904	0.024 *	1.626, 0.788–2.99	0.208	0.896, 0.529–1.624	0.790	-	-
**Sexual frequency**								
0–2 times weekly	reference		-	-	reference		-	-
3–5 times weekly	0.873, 0.554–1.375	0.559	-	-	1.054, 0.646–1.720	0.832	-	-
**Douching**								
No	reference		reference		reference		-	-
Yes	1.299, 1.079–2.740	0.023 *	1.321, 0.763–2.211	0.334	1.115, 0.668–1.862	0.677	-	-
**Smoker**								
No	reference		-	-	reference		-	-
Yes	1.182, 0.453–3.084	0.732	-	-	0.794, 0.257–2.456	0.689	-	-

Abbreviations: AOR: Adjusted odds ratio, COR: Crude odds ratio, 95% CI: 95% Confidence interval, (variables with a *P* value of less than 0.2 at bivariate analysis were entered into the multivariable analysis model).* Indicates significant association (*P* < 0.05)

## Discussion

In the present study, the prevalence of infectious vaginitis (58.5%) was high; however, it was comparable to 56.4% among pregnant women in Ghana [15]. Nonetheless, it was higher than the prevalence reported in India (46.96%) [21], Ethiopia 15.4% [1] and Iran (43.6%) [8]. However, a study done in Nigeria (76%), [14], a higher prevalence was reported in comparison to our study. Arguably, the variations are largely due to physiological status, population diversity, geographic location, ethnicity, socioeconomic factors, and varying sampling and culturing techniques [2, 6]. The high prevalence in this study could be attributed to the reported high frequency of sex and, with multiple partners, engaging in unprotected sex, since only 7.48% used condoms. Unprotected sex disrupts the normally acidic vaginal milieu due to the repeated alkalization by semen thus predisposing the vagina to infections [13]. Unprotected sex also increases the likelihood of acquiring other STDs, including HIV; therefore, there is a need for public education on the use of protected sex to prevent and reduce the risk of contracting any type of STD.

Generally, VVC was the more prevalent (33.2%) cause of infectious vaginitis in this study than BV (24.9%) or TV (0.28%). Similar trends were reported in Ghana, with a higher prevalence of VVC than BV: 36.5% vs. 30.9%, and in India; 30% vs. 17.3% [2, 15]. However, contrasting findings of higher prevalence of BV than VVC; 22% vs. 20% in Brazil and, 38% vs. 36% in Nigeria [14, 22] have also been reported. This variability in the trend of infection is due to the differences in populations studied, and indeed, it’s been shown to vary from country to country, from one region to another within the same country, [2] and by type of health facility [6].

Specifically, the prevalence of VVC (33.24%) in our study was comparable to findings from studies in Nigeria 36%, [14], and Ghana 36.5% [15]. Other studies found a lower prevalence, including Nigeria 28%% [23], India 17.3% [2], Ethiopia 8.3% [1], and Yemen 6.6% [5]. Factors like douching and recent antibiotic use, which are linked to VVC infection, could have contributed to the high prevalence in this study. In the present study, a third of the women douched regularly (30.47%) and had used antibiotics recently (36.74%), and indeed, both factors (douching: antibiotic use) (COR: 1.719, 95% CI: 1.079–2.740; COR: 1.592, 95% CI: 1.016–2.494) were significantly associated with VVC at bivariate analysis. While several douching substances alter the pH of the vagina, the lethal effect of antibiotics inhibits the growth of the normal vaginal micro-flora, which can cause severe depletion of lactobacilli species, leading to the proliferation of VVC [10, 15].

As regards the prevalence of BV, the current study reports a BV prevalence of 24.9%, which is comparable to studies in Yemen 27.2% [5] and Ethiopia 20.1% [24]. There was a lower prevalence reported from Cameroon 17.1% [13], Iran 15.2% [8], Nigeria 17.3 [25] South Africa (17.7%) [26], and Ethiopia (18.0% and 19.4%) ( [27, 28]). Studies in Nigeria, 38% [14] Ethiopia 48.6% [29], India 54.3% [21] and Ghana 30.9% [15] all reported higher prevalence rates. Evidently, the prevalence of BV is highly variable with prevalence ranging from 8–51.% depending on the population studied [5]. Differences in the methodological design, characteristics of study participants, diagnostic techniques applied, and geographical locales [2] can explain the variations.

The prevalence of TV in this present study is 0.28% (95% CI: 0.0004–0.0196), which was comparable to similar studies in Yemen (0.9%) [5], India (1.8%) [2], and in Ethiopia (2.1%) [1]. In contrast, in another study, a prevalence of 11.2.% was reported among females in Iran [8] however, there was a difference in the study design and diagnostic methods used. In the present study, trichomoniasis was diagnosed using a wet mount microscopy which has a low sensitivity. This could have contributed to the low prevalence of trichomoniasis. This is in contrast to the earlier studies in Uganda [16] that reported a higher prevalence of trichomoniasis (23.8%) because of the use of more sensitive diagnostic methods.

With regard to predictors of infection, pruritus was the only factor identified as an independent predictor of VVC (AOR: 2.861 (95% CI: 1.684–4.863), in the multivariable regression analysis model. Notably, women who presented with pruritus were 2.861 times more likely to have VVC than their counterparts. A similar finding was reported in an earlier study in Uganda [30]. Likewise, other studies in Nepal [31], Greece [4], and Yemen [5] are in concurrence. Among the symptoms of VVC, pruritus (itchiness) is noted as the most notorious symptom of VVC [10]. This clinical manifestation suggests a strong predictive clinical sign that could guide clinicians to reach a tentative diagnosis, especially in rural clinics in sub-Saharan Africa that lack appropriate diagnostic tools.

In this present study, in bivariate analysis, factors including higher education status, pregnancy, antibiotic use, douching, and multiple partners were associated with VVC, while factors like smelly discharge, IUD, and antibiotic use were associated with BV. However, the multivariable regression analysis model did not show statistical significance. In congruence, the authors Konadu et al. [15] found no statistical significance with either BV or VVC for factors including education, contraceptive use, douching, antibiotic use, and sexual partners in their study done in Ghana, despite multiple studies linking these variables to infection [5, 9, 29]. This suggests that several factors associated with infectious vaginitis vary with socioeconomic and demographic environments, population structure, and others.

This study provides primary data on infectious vaginitis among reproductive-age women seeking reproductive health services at a sexual and reproductive health facility in Kampala, Uganda. The application of two extremely sensitive diagnostic techniques the Nugent score and culture for the identification of BV and VVC, respectively, were some of the strengths of this study. Both diagnostic techniques are considered the gold standard tests for their respective infections [10, 32], and they are easily reproducible.

Notable limitations in this study include the use of a less sensitive method, wet-mount microscopy, for diagnosing trichomoniasis, whose sensitivity can be as low as 36% [10], and the choice of study design (cross-sectional), which precluded drawing conclusions about predictors of infection. Therefore, case-control studies are recommended to properly investigate the associations between infection and disease predictors, in addition to the use of more sensitive diagnostic tests to determine the true prevalence of trichomoniasis.

## Conclusions

The prevalence of infectious vaginitis is high among reproductive-age women seeking reproductive services at Marie-stopes health facility in Kampala, Uganda. Vulvo-vaginal candidiasis was the most prevalent, followed by bacterial vaginosis and trichomoniasis, respectively. Pruritus was the only factor significantly associated with infection. Regular screening and treatment of reproductive-aged women seeking reproductive health services would greatly reduce the prevalence of vaginitis infection. Although the results of this study may not be generalizable to the whole urban area of Kampala, it provides a preliminary understanding of the burden. Future studies ought to focus on the entire Kampala to understand the prevalence and risk factors.

## Data Availability

All the data generated and analyzed during this study are included in the manuscript. The corresponding author can provide original data supporting these findings upon reasonable request.

## Abbreviations

AOR: Adjusted Odds Ratio
BV: Bacterial Vaginosis
COR: Crude Odds Ratio
CI: Confidence interval
HIV: Human immunodeficiency virus
IUD: Intrauterine device
SDA: Sabouraud dextrose agar
STD: Sexually transmitted disease
TV: Trichomoniasis
VVC: Vulvo Vaginal Candidiasis
